# Children mirror adults for the worse: evidence of suicide rates due to air pollution and unemployment

**DOI:** 10.1186/s12889-022-14013-y

**Published:** 2022-08-25

**Authors:** Akihiro Okuyama, Sunbin Yoo, Shunsuke Managi

**Affiliations:** Urban Institute, School of Engineering, Kyushu University, Fukuoka, Japan

**Keywords:** Public health, Children suicides, Suicides, Pandemic

## Abstract

**Background:**

Every year, more than 700,000 people die due to suicide, one of the most common reasons for youth death. While many studies have revealed two main factors for suicidal behavior: impulsive suicidal behavior due to mental illness and financial stress, it is not clear what happens if individuals face deterioration of mental health and economic recession. This paper attempts to answer this question and how suicide rates are correlated with these factors.

**Methods:**

We empirically investigate whether economic recessions and air pollution trigger suicides by examining Japan, a country with one of the highest suicide rates, from 2014 to 2021. We take advantage of the characteristics of the COVID-19 pandemic and the periods before the pandemic, when both economic recessions and reductions in air pollution occurred simultaneously. Using monthly and municipal- level data, we construct a triple difference model that takes air pollution and unemployment as treatments.

**Results:**

Our findings show that high (upper half of each period) levels of air pollution and unemployment have substantial impacts on the suicide rates of adults (22.9% in the short term) and children (42.7% in the short term, 36.0% in the long term), indicating that the increase in suicide rates among children is almost twice as high as that among adults. Our study finds that unemployment and air pollution alone are not associated with increased suicide rates but their simultaneous occurrence triggers suicides during the pandemic.

**Conclusions:**

Our study urges suicide prevention, particularly among children, as an essential consideration for public health. Furthermore, our results indicate the need for the government to allocate resources to recover air quality and the economy simultaneously during a recession to reduce suicide mortality of both child and adults.

**Supplementary Information:**

The online version contains supplementary material available at 10.1186/s12889-022-14013-y.

## Introduction

Suicide is one of the most common reasons for youth death (age 15–29), resulting in 700,000 deaths annually–and indeed, in addition to the number of suicide victims, there are vast numbers of people who attempt to kill themselves [1]. A large number of studies have found that the two dominant reasons for suicidal behaviors are impulsive suicidal attempts/behaviors due to mental illness [2–5] and financial stress [6–8]. Therefore, what happens if individuals face a recession and exacerbation of the mental illness simultaneously? This study attempts to answer this question and determine how suicide rates are associated with these variables.

While observing financial stress is relatively simple, as we can examine macroeconomic indices (i.e., income levels, GDP, or unemployment rates), tracking individual level data on mental illness and suicidal attempts is challenging. To overcome this problem, we choose to employ air pollution concentration as a proxy of one of the dominant factors that triggers suicidal behaviors by aggravating mental illness. We refer to previous research [9–15] showing that air pollution is positively associated with impulsive suicidal behaviors. We also refer to previous works showing that such trends are stronger for people with a mental illness and children [7, 16–19].

The background provided by previous works allows us to provide quantitative evidence on the impacts of the economic downturn (represented as unemployment growth) and the increased likelihood of the induction of impulsive suicidal behaviors (triggered by air pollution).^1^

To this end, we begin by establishing our empirical framework. First, we use air pollution and unemployment rates as two main independent variables in our empirical framework. Second, we set our study period from 2014 to 2021. Therefore, our study periods cover the COVID-19 pandemic, which has limited people’s (economic) activities and reduced air pollution. The pandemic provides a natural experimental condition that allows us to examine both reduction of the air pollution and the unemployment growth at the same time. Notably, our study period also includes a period before the COVID-19 pandemic. Therefore the implications from our study can be extended beyond the pandemic situation. Finally, we analyze the impact of air pollution reductions and unemployment growth on diverse subpopulations divided by gender and age group.

**Fig. 1:**
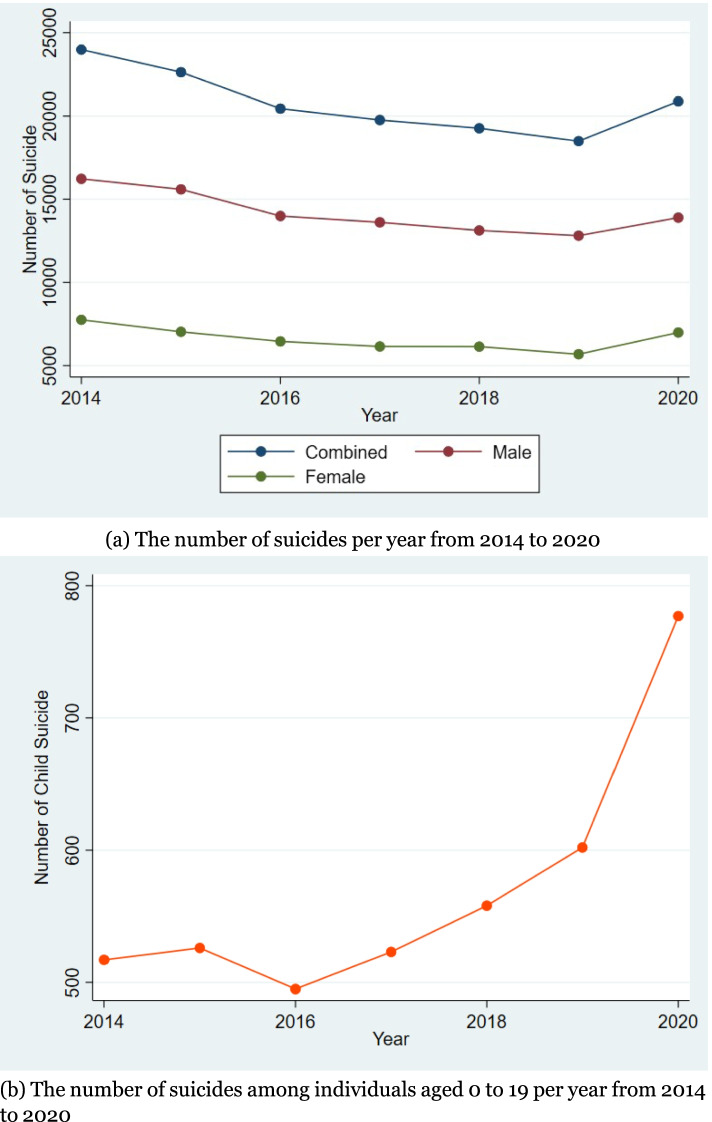
Suicide Trend. **a** The number of suicides per year from 2014 to 2020. **b** The number of suicides among individuals aged 0 to 19 per year from 2014 to 2020

We choose Japan for the following three reasons. First, suicide is one of the main causes of death in Japan, particularly for Japanese people aged between 15 and 39. Such high suicide rates make Japan the only G7 country where suicide is the leading cause of death for young people.^2^ The suicide rate in Japan reached the peak in 2020, with the rates increasing for the entire population, women, and people aged 0 to 19 by approximately 4%, 15%, and 44%, respectively (Figure 1). Second, the Japanese economy is experiencing a historically unprecedented recession during the COVID-19 pandemic. The Japanese GDP declined by 4.6% in 2020. Moreover, many people have started to lose their jobs. The annual unemployment rate increased (by 0.4%) in 2020, and this increase has been for the first time since 2009 (Lehman shock). The rise of the unemployment rate could increase the level of extreme stress for Japanese because lifetime employment is regarded as the standard for Japanese people [20]. The year 2020 marked the first time young people were concerned about their occupation since the time they started working. Third, air pollution has been alleviated in Japan during the COVID-19 pandemic due to the stay-home order and decrease in business activities. In Japan, the surface PM_2*.*5_ concentration decreased by 30%-50% during February and March 2020 compared to that in the same period in 2018 and 2019 [21].

To this end, we build a triple difference model by using monthly and municipality level data on suicide rates categorized by gender, age group, air quality data, and data on unemployment rates covering the entire Japanese population in 47 municipalities from 2014 to 2021. Building a triple difference model requires the treatment variables to be independent. Notably, we focus on the entire COVID-19 pandemic period rather than a specific lockdown period. Therefore, it is less likely that the start of the COVID-19 pandemic is correlated with the rapid increase in unemployment rates and reductions in air pollution. This research setting allows us to eliminate possible concerns about endogeneity, for example, lockdown causing the growth of unemployment. We examine the short-term effects of the pandemic by examining the first waves of pandemic (February- May 2020) and the long-term effects by examining the second waves (July-September 2020).

## Background

### Literature review and our contribution

Many previous studies have revealed that ambient air pollution [22–27] and economic recession [28, 29] trigger suicide. For example, teenagers (younger than 18) have a more than 10 times higher risk of suicide-related emergency ambulance dispatches for psychiatric emergencies after exposure to PM_2*.*5_ than adults (age between 18 and 64) [30]. Regarding economic recession, [29] report a change in the child suicide rate after Great Recession in 2007.

Our study is not the first to examine the association between economic recession, air pollution, and suicide rate. However, no study has examined both of them simultaneously. In this study, we try to decompose the impact of air pollution and economic recession on the suicide rate. By doing so, we can determine which factor has a stronger negative impact on children’s mental health, which would be helpful to design policy measures to protect people from suicidal behavior.

Several studies have examined the trend of the suicide rate during the COVID-19 pandemic [31, 32], and a few studies have investigated the impact of air quality and economic conditions during pandemic on the suicide rate. During the global COVID-19 pandemic, air quality in many areas has improved [33], although the pandemic has had a severe negative impact on the global economy (according to International Monetary Fund, the growth in the real-world gross domestic product (GDP) decreased by 3.3% in 2020 compared to that 2019 [34]. Therefore, it is not clear whether the number of suicides decreased during the pandemic due to the reduction of air pollutants or increased because of economic recession. Thus, we quantify the impacts of economic recession and air pollution on the suicide rate during the COVID-19 pandemic.

Several studies have shown the impact of air pollution and economic recession on children’s psychological and physical health by focusing on hospital visits [30, 35, 36].

The findings of these works on the increase in hospital visits are worth examining. However, we identify a research gap because the increase in hospital visits does not indicate that air pollution and recession are adversely associated with the children’s health, as they might merely show an increase in the number of hospital visits. Thus, we choose to examine the suicide rates of children, which represent an obviously adverse health outcome. Therefore, to resolve this gap, we scrutinize the relationship between the suicide rate, air pollution, and recession to examine whether the latter two variables lead to serious health problems. To this end, we start from the previous works [16, 18, 37] show that children are more vulnerable to air pollution, as they are more likely to behave impulsively, which results in suicidal behaviors.

### Unexpected outcomes of social distancing

Previous studies have shown the effectiveness of social distancing in diverse aspects. Yoo and Managi [38], Ferguson et al. [39], Greenstone and Nigam [40] express the effectiveness of social distancing in suppressing infections in terms of monetary value. However, other strands of literature have shown that social distancing, which includes case isolation and quarantine, triggered mental depression and anxiety during the SARS epidemic [41]. While it is evident that depression is critically connected to suicide attempts, the question of whether social distancing is also interconnected with suicide rates has not yet been clearly investigated. Thus, we contribute to the literature by showing evidence on the *unexpected outcomes* of social distancing that may increase the risk of suicides.

In this study, we additionally examine teenagers because some literature has pointed out the negative impact of social distancing on teenagers’ mental health [42]. Given that depression is closely connected to suicide attempts, social distancing might have a positive impact on suicide growth (especially for children).

**Fig. 2:**
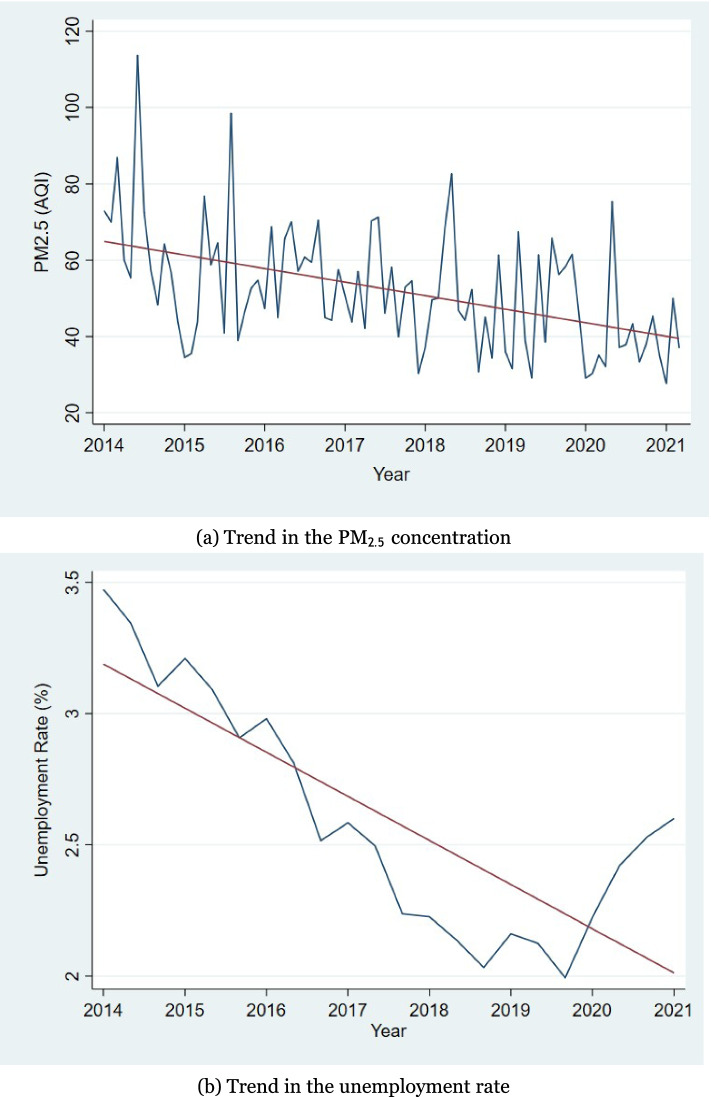
Change in the concentration of PM_2*.*5_ and unemployment rate over time. **a** Trend in the PM_2*.*5_ concentration. **b** Trend in the unemployment rate

Because we employ a triple difference approach, which requires a randomized controlled group (thus, the treatment groups should not have correlations), we first provide graphical evidence that shows the absence of a correlation between air pollution and unemployment rates during the first and second waves of the pandemic. Figure 2 shows the time trend of PM_2*.*5_ and unemployment rate. Panel (a) presents the change in the average concentration of PM_2*.*5_ over time in Japan. While it fluctuates starting in 2014, it starts to show a gradual decrease from a value of approximately 60 on the air quality index. On the other hand, panel (b) shows the change over time in unemployment in Japan during the study period. The unemployment trend shows a gradual decline from 2014 until 2019. However, the unemployment rate increases starting in 2020. Compared to Panel (a) and (b), during the first wave of the COVID-19 pandemic, the PM_2*.*5_ concentration fluctuates from 30.3 to 75.4, while the unemployment rate continues to increase during this period. In the second wave, the concentration of PM_2*.*5_ increases from 37.8 to 43.3 and immediately drops to 33.4. However, the unemployment rate continues to increase from 1.99.

Second, we present the correlation matrix of unemployment rate and concentration value of PM_2*.*5_ (Table 1). The correlation coefficient between unemployment rate and PM_2*.*5_ is 0.1994 during the first wave and 0.060 during the second wave. This indicates that there is only a weak statistical correlation between unemployment and PM_2*.*5_.

**Table 1:** Pearson correlation matrix

Variables	UE (1st wave)	PM_2*.*5_ (1st wave)	UE (2nd wave)	PM_2*.*5_ (2nd wave)
UE (1st wave)	1.000			
PM_2*.*5_ (1st wave)	0.1994	1.000		
UE (2nd wave)			1.000	
PM_2*.*5_ (2nd wave)			0.060	1.000

Note that *UE* is abbreviation for unemployment rate

Both the graphical evidence and correlation matrix show that the correlation between the air pollution and the unemployment rates is unlikely to exist, and thus, we can conduct empirical analysis using a triple differences approach.

### Situations in Japan during the COVID-19 pandemic and policy

**Fig. 3:**
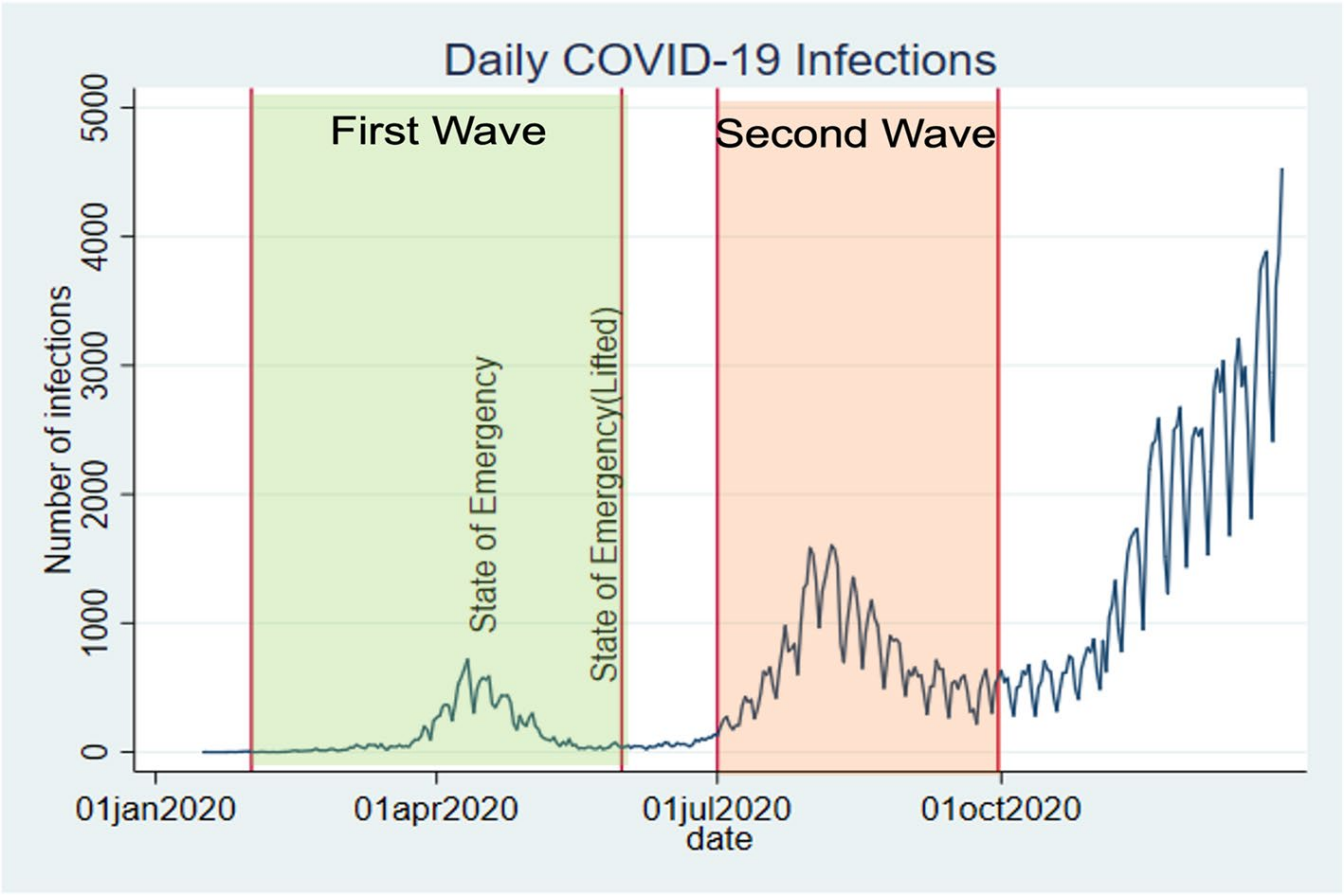
Number of cases in Japan

The Japanese government confirmed the country’s first cases of COVID-19 on 16 January 2020 among people returning from Wuhan, China. Although the government made an effort to prevent the spread of infections, the number of cases continued to increase (Figure 3). On February 27, the prime minister requested school closures from March 2 to the beginning of April. On March 27, the number of daily new cases reached over 100 for the first time. On April 7, the government declared a state of emergency until May 6 for seven prefectures in severe situations, including Tokyo. As the number of infections increased, the government expanded the state of emergency to all 47 prefectures within the country on April 16. The state of emergency required people to stay at home and work from home. It also requested that many stores and schools be closed (e.g., restaurants, gyms, department stores). During the state of emergency, the number of new daily confirmed cases was at a record high for the nation of 588 cases. The state of emergency was lifted for the whole country on May 25. At this point, the number of new daily COVID-19 cases remained low; the number of daily new confirmed cases was smaller than 100.

The request for residents of four prefectures, including Tokyo, to refrain from leaving the prefecture was lifted on June 19. However, COVID-19 started spreading again from the end of June. The number of new cases per day remained over 100. Despite the spread of infectious disease, the national government did not implement a health intervention as severe as a state of emergency. Instead, the municipal government of particular prefectures declared prefecture-specific states of emergency. On August 7, 1,605 cases were confirmed, which was the highest daily record. The number of infections subsequently started decreasing until the end of September. In our analysis, we divide the COVID-19 pandemic into two phases. We define the period between February 2020 and May 2020 as the first wave and the period from July 2020 to September 2020 as the second wave. By separating the pandemic phases, we can investigate the initial and short-term influence of the pandemic on the suicide rate as well as the long-term effect of the pandemic on the suicide rate.

## Empirical strategy

### Data

In this section, we introduce three datasets used in this study. First, we collect data on the number of suicides divided by sociodemographic information in Japan. Second, we use the PM_2*.*5_ air quality index (AQI), which shows the concentration of air pollutants and their effect on people’s health. Third, we employ the data on the unemployment rate, which represent economic situations such as recessions.

After matching these datasets according to prefecture, year, and month, we merge them into one dataset and proceed to our empirical analysis. Consequently, we acquire 3,995 from 47 prefectures from 2014 January to 2021 March.

#### Data on suicide

The Ministry of Health, Labor, and Welfare provides data on suicides in Japan since January 1, 2006, and we collect the data from January 1, 2014, to March 31, 2021 [43].

This aggregated dataset includes information such as the number of suicides by age, sex, employment status, site, and reason, as well as the number of attempted suicides. The dataset includes information on 150,668 suicides in 47 prefectures based on the numbers reported at the municipality level. Children and teenagers (aged between 0 and 19 years old) account for 2.77% of the total suicides, and adults (aged between 20 and 69 years old) make up 72.1% of this total.

#### Data on air pollution

Data on the concentration of particular matter with an aerodynamic diameter ≤ 2*.*5 µm (PM_2*.*5_) were acquired for the study period (January 1, 2014 - March 31, 2021) in all prefectures in Japan from the World Air Quality Index project [44]. The AQI is an index for reporting daily air quality based on the concentration of air pollutants. We use AQI data that are estimated through a method used by the US Environmental Protection Agency (USEPA) [45]. The AQI is an important index for the public to understand how good or bad the air quality is for their health. According to the USEPA, the AQI has an estimated value of 0–500 [46]. The higher the value of the AQI is, the greater the level of air pollution, and the higher the health risk. For instance, an AQI value smaller than 50 is categorized as good with little health risk.

#### Data on unemployment rate

We obtain data on the unemployment rate from the Monthly Labor Survey from the Statistic Bureau of Japan, which is provided by the Ministry of Health, Labor, and Welfare [47]. These data provide the quarterly average number of working and employed people and the unemployment rate in 47 different prefectures in Japan from January 1997 to March 2021.

### Study Design

Figure 2 demonstrates that the COVID-19 pandemic does not directly affect the PM_2*.*5_ concentration or the unemployment rate. The concentration of PM_2*.*5_ continues to fluctuate after the outbreak of COVID-19, similar to the previous period. In addition, the unemployment rate begins to rise at the end of 2019 (before the government confirmed the first case of COVID-19 infections). Even though the unemployment rate continues to increase, the slope of a line stays the same as that at the end of 2019. Therefore, there is no direct impact of the COVID-19 pandemic on the unemployment rate. Moreover, since we focus not on the lockdown or COVID-19 itself but on the COVID-19 pandemic phase, we assume that we can conduct empirical analysis about the unemployment rate and air pollution during the COVID-19 pandemic. Therefore, these facts validate our use of triple difference analysis (eq. 1).

1 $${Y}_{jym}=\alpha +{\beta }_{1}{First}_{ym}+ {\beta }_{2}{Second}_{ym}+{\beta }_{3}{AP}_{jym}+{\beta }_{4}{UE}_{jym}+ {\beta }_{5}\left({First}_{ym}\times {AP}_{jym}\right)+{\beta }_{6}\left({First}_{ym}\times {UE}_{jym}\right)+{\beta }_{7}\left({Second}_{ym}\times {AP}_{jym}\right)+{\beta }_{8}\left({Second}_{ym}\times {UE}_{jym}\right)+{\beta }_{9}\left({First}_{ym }\times {AP}_{jym} \times {UE}_{jym}\right)+{\beta }_{10}\left({Second}_{ym}\times {AP}_{jym}\times {UE}_{jym}\right)+{\zeta }_{j}+{\xi }_{y}+{\psi }_{m}+{\mu }_{jm} +{\gamma }_{jy}+{\epsilon }_{jym}$$

where *Y*_*jym*_ is a logged suicide rate of prefecture *j* in month *m* in year *y*. We employ six de pendent variables according to age and gender. These six dependent variables are com parable because the data source and independent variables we use are the same for all dependent variables. *First*_*ym*_ is a dummy variable that takes a value of 1 if the periods of observations correspond to February 2020 to May 2020. *Second*_*ym*_ is also a dummy variable that takes a value of 1 from July 2020 to September 2020. *AP*_*jym*_ is a dummy variable that takes a value of 1 if the AQI PM2.5 value is in the top 50th percentile of each period.

It takes 1 if the AQI is 50 or more during the periods except for the first and second waves, or if the AQI is 42 or more during the first wave, and 38 or more during the second wave. *UE*_*jym*_ is a dummy valuable that takes a value of 1 if the unemployment rate is in the top 50 percentile of respective periods. It takes 1 if the unemployment rate is 2.5 or more during the time except for the first and second waves, or if the unemployment rate is 2.2 or more during the first wave, and 2.4 or more during the second wave. In our model, *β*_9_ and *β*_10_ are the parameters of interest that denote the impact of the COVID-19 pandemic, air pollution, and unemployment on the suicide rate.

We include several types of fixed effects. First, we employ interaction terms with prefecture- month (*µ*_*jm*_) and prefecture-year (*γ*_*jy*_), which controls for yearly and monthly-specific shocks, respectively, in each prefecture, such as seasonality in the suicide rate, monthly local events, and climatic conditions. By including yearly interactions with the prefecture, we can control for macroeconomic trends, industrial or population structural changes, or suicide trends of each prefecture. Second, we include prefecture (*ζ*_*j*_), year (*ξ*_*y*_) and monthly (*ψ*_*m*_) dummy variables. All methods were carried out in accordance with relevant guidelines and regulations.

## Result

Table 2 represents the results of the triple difference model, and the main results of this study.^3^ All the independent variables we use in this study are dummy variables. The first to sixth columns refer to the relationship between dependent variables (suicide rate of adults, children, male adults, male children, female adults, and female children) and each independent variable. The interpretation of the results can be made as follows: the coefficient of column (1) in the first row is -0.0911, revealing that the logarithmic value of the adult suicide rate during the first wave decreases by 9.11%. The coefficients of the tenth and eleventh rows are our main parameters of interest.

**Table 2:** Triple difference estimation

	(1)	(2)	(3)	(4)	(5)	(6)
	Adult SR	Child SR	Male Adult SR	Male Child SR	Female Adult SR	Female Child SR
First	-0.0911 ∗	-0.1249	-0.0158	-0.0397	-0.2656 ∗ ∗ ∗	-0.2139
	(0.0509)	(0.1278)	(0.0589)	(0.1335)	(0.0870)	(0.1687)
Second	-0.0041	0.1407	0.0075	0.0413	0.1317	-0.0695
	(0.0556)	(0.1162)	(0.0644)	(0.1191)	(0.0974)	(0.1301)
AP	0.0041	0.0076	-0.0036	-0.0266	-0.0141	0.0428
	(0.0175)	(0.0409)	(0.0203)	(0.0431)	(0.0296)	(0.0505)
UE	0.0322 ∗	0.0308	0.0011	-0.0294	0.0636 ∗ ∗ ∗	0.0215
	(0.0179)	(0.0401)	(0.0207)	(0.0425)	(0.0302)	(0.0480)
AP × UE	-0.0067	-0.0490	0.0150	0.0130	-0.0022	-0.0817
	(0.0210)	(0.0468)	(0.0243)	(0.0489)	(0.0352)	(0.0569)
First × AP	-0.1300 ∗	-0.1069	-0.1601 ∗ ∗ ∗	-0.2861	-0.0473	0.1481
	(0.0675)	(0.1663)	(0.0781)	(0.1827)	(0.1147)	(0.1995)
Second × AP	0.0560	-0.2667	0.1090	-0.0603	-0.1384	-0.1941
	(0.0742)	(0.1674)	(0.0859)	(0.1799)	(0.1279)	(0.2017)
First × UE	-0.2244 ∗ ∗ ∗	-0.1557	-0.1925 ∗ ∗ ∗	-0.1417	-0.2059 ∗	0.0233
	(0.0633)	(0.1565)	(0.0733)	(0.1699)	(0.1081)	(0.1939)
Second × UE	0.0404	0.0430	0.0080	0.0415	0.0066	0.2451
	(0.0732)	(0.1501)	(0.0847)	(0.1508)	(0.1245)	(0.1574)
First × AP × UE	0.2291 ∗ ∗ ∗	0.4266 ∗ ∗ ∗	0.2406 ∗ ∗ ∗	0.5870 ∗ ∗ ∗	0.1673	0.1170
	(0.0893)	(0.2075)	(0.1033)	(0.2289)	(0.1518)	(0.2411)
Second × AP × UE	-0.0091	0.3596 ∗	-0.0412	0.2067	0.0526	0.0801
	(0.1018)	(0.2125)	(0.1179)	(0.2214)	(0.1715)	(0.2367)
_cons	-11.0599 ∗ ∗ ∗	-12.5613 ∗ ∗ ∗	-10.6875 ∗ ∗ ∗	-12.0909 ∗ ∗ ∗	-11.6926 ∗ ∗ ∗	-12.2826 ∗ ∗ ∗
	(0.0124)	(0.0300)	(0.0144)	(0.0323)	(0.0213)	(0.0376)
*Observations*	995	2014	3992	1620	3813	1000
*R*^*2*^	0.246	0.565	0.226	0.674	0.119	0.784

Standard errors in parentheses

^*^^p < 0.1, ** *p* < 0.05, *** *p* < 0.01^

Adult SR: log of suicide rate among adults

Child SR: log of suicide rate among children

Male Adult SR: log of suicide rate among male adults

Male Child SR: log of suicide rate among male children

Female Adult SR: log of suicide rate among female adults

Female Child SR: log of suicide rate among female children

Our results indicate that the interaction between air pollution and economic recession increases suicide rates. During the first wave, suicide rates among adults and children increase by 22.91% and 42.66%, respectively, if they lived in areas with high unemployment and expose to high air pollution. This shows that the increase in suicide rates among children is double that of adults. Furthermore, males are more vulnerable than females. The suicide rates among male adults and children increase by 24.06% and 58.70%, respectively, while suicide rates among females show a nonsignificant change.

During the second wave, the suicide rate among children increases by 35.96% for those living in areas experiencing high unemployment rates and air pollution.

## Discussion

Our study shows the importance of focusing on vulnerabilities associated with exposure to both air pollution and unemployment. Air pollution and economic recession (increase of unemployment rate) are positively associated to psychological issues [48–51]. These negative effects of air pollution and recession on individuals’ mental health could trigger suicide. Our result supports that unemployment is positively associated with adult suicide rates, and we confirm that suicide rates temporarily decreased if a person was unemployed during the first wave. On the other hand, our results do not show a positive interplay between exposure to high air pollution and suicide rates; again, our results show that during the first wave, suicide rates decreased in the regions where air pollution concentration was high. Nevertheless, we provide clear empirical evidence that a high unemployment rate and air pollution increased the suicide rate if they occurred simultaneously during the pandemic.

This study enables policymakers to move beyond simply preventing suicides caused by dominant factors to actively monitor those who are unemployed as well as exposed to air pollution. Our results inspire the design of effective policy instruments that prevent suicides. Even though our research is not based on individual-level data, it is still helpful for policymakers. Our results indicate that first wave dummy variables are negatively correlated with the suicide rate of several groups, which is consistent with the previous study [52]. In addition, much research suggests that suicide deaths decreased in the initial stage of COVID-19 outbreaks in many other countries such as Norway, the UK [53], Germany, and Peru [54–56]. Therefore, the decrease in the suicide rate in the initial stage of the public health crisis is not surprising.

### Child suicide rate

Our results show that air pollution and unemployment rates alone do not have any relationship with the child suicide rate. However, a high unemployment rate and high air pollution increase the child suicide rate if they happen simultaneously. We employ air pollution and unemployment as proxies for psychological health issues and economic recession. Therefore, our results indicate that children are vulnerable to psychological health issues and economic recession. Our findings show that high air pollution and unemployment rates increased the child suicide rate (42.66% in the short term, 35.96% in the long term) and the male child suicide rate (58.7% in the short term). Our results show that the increase in the child suicide rate was higher than the increase in the adult suicide rate (22.91% in the short term). There are several possible reasons why children were more vulnerable to the combination of high air pollution and unemployment in the first wave. Strict stay-at-home orders during the first wave may have impacted children’s physiological health. Unfortunately, the data limitations do not allow us to investigate this mechanism. To discover the reasons behind this result, we need detailed psychological and physical health reports on each suicide victim to understand why they died by suicide. Therefore, examining why the combination of air pollution and unemployment increases the child suicide rate more than the adult suicide rate is worth examining in future research. Although we cannot identify the mechanism, our study is the first to find that the combination of high air pollution and unemployment increases the child suicide rate.

### Adult suicide rate

**Fig. 4:**
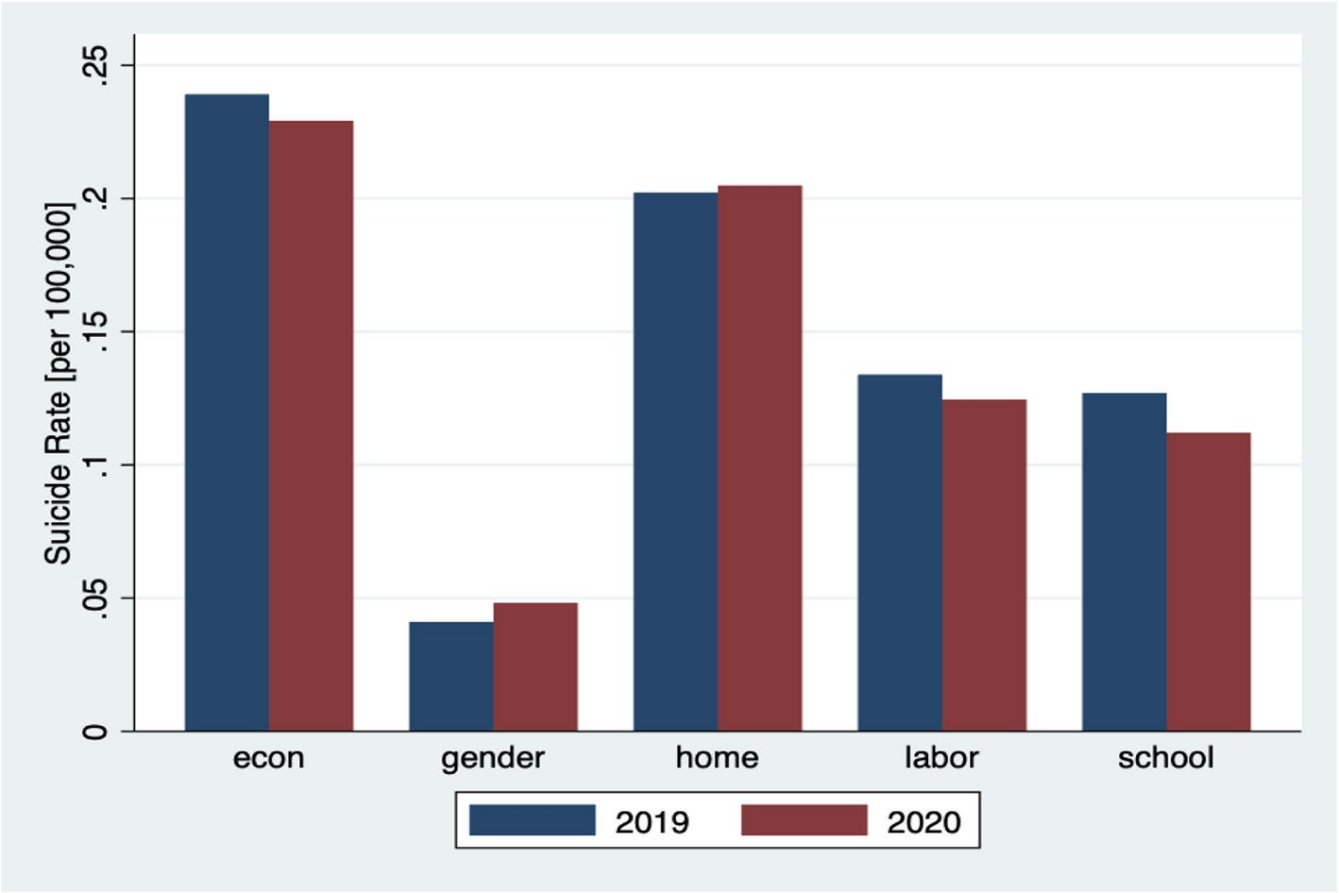
Suicide rate by reason between February and May in 2019 and 2020

We find that high unemployment in the first wave decreased the suicide rates among adults, male adults, and female adults by 22.44%, 19.25%, and 20.59%, respectively. Even though there are complex reasons behind suicide, there are many possibilities that could explain our results for adults. The reason why the high unemployment rate decreased the suicide rate during the first wave can be partially explained by emergency subsidies provided by the Japanese government. Approximately 80% of cash was distributed to all citizens (all Japanese people were eligible to gain 100,000 yen (940 USD)) by June. This cash benefit may have relieved financial stress for Japanese people. In addition, the average suicide rate due to economic and financial problems and labor problems decreased from February to May 2019 to February to May 2020, while the suicide rate due to other reasons increased from 2019 to 2020 (Figure 4).

During the first wave, we find that the suicide rate for adults, especially males, decreased by 13.00% and 16.01%, respectively, with exposure to higher air pollution. This may be because of the strict stay-home orders from the Japanese government during the first wave. On April 7, the government declared a state of emergency as of May 6 for seven prefectures experiencing severe situations, including Tokyo. As the number of infections increased, the government expanded the state of emergency to all 47 prefectures within the country on April 16. The state of emergency required people to stay at home and work from home. It also requested that many stores and schools to be closed (e.g., restaurants, gyms, department stores). During the first wave, many workers worked from home and many companies request that their employees reduce their commuting days. This may have reduced the suicide rate among adults and male adults even if the air pollution concentration was high during the first wave. We infer that ambient air pollution does not deteriorate people’s mental health compared with that during the pre-COVID-19 period because during the first wave, people, especially adults and male adults who were employed, spent less time outdoors.

Our results show that during the first wave, high air pollution or high unemployment alone decreased adult suicide rates, while the combination of both increased adult suicide rates. We cannot analyze the reason behind these results due to our data limitation, although it is worthwhile to understand this mechanism. However, our study provides important implications for children and future policies by showing that the simultaneous occurrence of high air pollution and a high unemployment rate trigger suicide.

We also find that a higher unemployment rate increased the suicide rate among adults, especially female adults, by 3.22% and 6.36%, respectively, which is consistent with previous work that suggests the rates of suicide attempts of unemployed women are higher than those of employed women [57]. Moreover, female adults may be more likely to experience psychological harm when they are unemployed than male adults because the number of employed women is smaller than that of male workers in Japan.^4^

### Government’s measures’

Given that a higher level of air pollution indicates a higher level of economic activity, the results of this research provide supporting evidence that social and governance measures may produce ‘unexpected’ outcomes [58]. We find that the suicide rate among children and adults increased when the unemployment rate and level of air pollution were high during the first wave. This finding indicates that the suicide rate could increase when government measures are strict and the unemployment rate is high while the level of economic activity is still high. However, our triple difference estimations show that the suicide rate does not change when economic activity is accelerated and the unemployment rate is high. This reveals that suicide rates do not increase even if the unemployment rate and the level of economic activity are high without government measures.

Therefore, strict government measures, such as lockdowns or the declaration of a state of emergency could increase the suicide rate significantly during an economic recession if the level of economic activity is high.

### Policy implication

Our results suggest some policy implications for preventing suicidal behaviors. First, our results provide evidence of the need for air quality control during the recession to prevent suicide. Our study shows that economic recession solely decreases the suicide rate during recession. However, if a recession is accompanied by air pollution, the impact of the recession on the suicide rate becomes significantly positive. This result indicates the need for the government to allocate resources to recover air quality and the economy simultaneously during a recession to reduce suicide mortality.

Our results suggest to policymakers the need for suicide prevention for children during the COVID-19 pandemic. We find that the impact of the COVID-19 pandemic on the child suicide rate is long term. Thus, the government should implement a policy to protect children from suicidal behavior because there are no signs that the situation is being brought under control in Japan. Previous studies have suggested several ways to protect children from suicide [59, 60]. To protect children from suicide mortality, the government should implement a policy to save children who cannot seek help during the pandemic.

We also identify suicide prevention needs for female adults. We find that an increase in the unemployment rate significantly increases the suicide rate among female adults.

We conjecture that Japanese females are more vulnerable than other populations to economic recession because Japanese women suffer from a considerable gender pay gap.^5^

This harmful gender gap for Japanese female adults may lead to suicide when they face mental deterioration due to recession. Therefore, improvement of women’s social status and a solution to the gender gap is needed to reduce suicide mortality for female adults.

## Conclusion

In this study, we examine whether economic recession and air pollution trigger suicide by investigating the suicide rate in Japan, where suicide is one of the main causes of death. Specifically, we configure our data period from 2014 to 2021 to cover the COVID- 19 pandemic period, when an economic recession and the improvement of air quality occurred simultaneously. The results of the triple difference model suggest that economic recession and air pollution trigger suicide among adults and children. Furthermore, we find that children were more likely to be vulnerable to economic recession and air pollution than adults during the first wave of the pandemic (the coefficient of the child suicide rate in the triple difference model is twice that of adults). In addition, our results of the triple difference model show positive and significant associations with the suicide rates of both children and adults during the first wave but a positive and significant relationship with the suicide rate of only children during the second wave. This finding proves that the impact of the COVID-19 pandemic is long term for children, while it is short term for adults.

While our identification strategy using triple difference models was appropriate, without detailed individual data (i.e., whether a person was suffering from depression), we cannot prove clear causality because our dataset is not at the individual level. The main reason for our inability to demonstrate causality is that we cannot scrutinize whether economic recession and air pollution immediately trigger suicide. Nonetheless, our study is still valid because we find a trend of economic recession and air pollution increasing suicide rates. Future research should focus on individual suicide attempts to address causality if individual-level suicide data are available.

## Supplementary Information

**Additional file 1.**

## Data Availability

The datasets generated and/or analyzed during the current study are available in the GitHub repository, https://github.com/akiokuyama/Children-Mirror-Adults-for-the-Worse/tree/main/data

## Abbreviations

AQI: Air Quality Index
GDP: Gross Domestic Product
G7: Group of Seven
DV: Domestic Violence
USEPA: United States Environmental Protection Agency

## Footnotes

1 The impact of air pollution on triggering suicides has been discussed in previous works; please refer to [22–26] for more details We further discuss this issue in Sect. 2.1.

2 Furthermore, the suicide rate itself is higher than that in other countries (16.3% in Japan, 7.4% in Germany, which has the lowest suicide rate among the G7 countries).

3 We additionally conduct difference-in-difference estimations to check the robustness of our main results and results are displayed in appendix.

4 [61] finds that women are more likely to be mentally vulnerable than men.

5 The global gender gap index ranked Japan 120 place out of 156 countries [62]
