# Supplementary material for: Children mirror adults for the worse: evidence of suicide rates due to air pollution and unemployment
Source: BMC Public Health. 2022 Aug 25;22:1614. doi: 10.1186/s12889-022-14013-y (PMC9403225; doi:10.1186/s12889-022-14013-y)
Supplement: Supplementary file 1 — Additional file 1. [file 12889_2022_14013_MOESM1_ESM.docx]

# Appendix

1. **Results of difference in difference**

We present the equation and results of difference-in-difference models in equation [1](#_bookmark0) and Table [A1](#_bookmark1).

*Y_jym_* = *α* + *β*_1_*First_ym_* + *β*_2_*Second_ym_* + *β*_3_*AP_jym_* + *β*_4_*UE_jym_*

+ *β*_5_(*First_ym_* × *AP_jym_*) + *β*_6_(*First_ym_* × *UE_jym_*) + *β*_7_(*Second_ym_* × *AP_jym_*)

+ *β*_8_(*Second_ym_* × *UE_jym_*) + *ζ_j_* + *ξ_y_* + *ψ_m_* + *µ_jm_* + *γ_jy_* + *ϵ_jym_*

(1)

Table A1: DID Estimation

(1) (2) (3) (4) (5) (6)

Adult SR Child SR Male Adult SR Male Child SR Female Adult SR Female Child SR First -0.1518∗∗∗ -0.2624∗∗∗ -0.0796 -0.2112∗ -0.3109∗∗∗ -0.2649∗∗∗

(0.0451) (0.1085) (0.0522) (0.1156) (0.0766) (0.1312)

Second -0.0012 0.0526 0.0184 -0.0071 0.1179 -0.0896

(0.0496) (0.1040) (0.0574) (0.1073) (0.0854) (0.1165)

AP -0.0020 -0.0127 -0.0094 -0.0463 -0.0198 0.0370

(0.0172) (0.0402) (0.0199) (0.0424) (0.0291) (0.0494)

UE 0.0279 0.0158 -0.0029 -0.0445 0.0593∗∗∗ 0.0173

(0.0177) (0.0397) (0.0205) (0.0420) (0.0298) (0.0474)

AP UE 0.0038 -0.0149 0.0249 0.0456 0.0075 -0.0722

×

(0.0202) (0.0450) (0.0234) (0.0471) (0.0338) (0.0542)

First AP 0.0009 0.1645 -0.0227 0.0868 0.0483 0.2274∗∗∗

×

(0.0442) (0.1004) (0.0512) (0.1103) (0.0751) (0.1133)

Second AP 0.0515 -0.0499 0.0880 0.0707 -0.1097 -0.1385

×

(0.0524) (0.1074) (0.0607) (0.1095) (0.0879) (0.1146)

First UE -0.1103∗∗∗ 0.0831 -0.0727 0.1760 -0.1219 0.0979

×

(0.0451) (0.1047) (0.0522) (0.1166) (0.0766) (0.1169)

Second UE 0.0351 0.2199∗∗∗ -0.0138 0.1365 0.0335 0.2803∗∗∗

×

(0.0515) (0.1079) (0.0595) (0.1124) (0.0866) (0.1198)

_cons -11.0579∗∗∗ -12.5544∗∗∗ -10.6857∗∗∗ -12.0837∗∗∗ -11.6906∗∗∗ -12.2804∗∗∗ (0.0124) (0.0299) (0.0143) (0.0322) (0.0212) (0.0374)

*Observations* 3995 2014 3992 1620 3813 1000

*R*^2^ 0.245 0.564 0.225 0.672 0.118 0.784

Standard errors in parentheses

∗ *p <* 0*.*1, ∗∗ *p <* 0*.*05, ∗∗∗ *p <* 0*.*01

Adult SR: log of suicide rate among adults Child SR: log of suicide rate among children

Male Adult SR: log of suicide rate among male adults Male Child SR: log of suicide rate among male children Female Adult SR: log of suicide rate among female adults

Female Child SR: log of suicide rate among female children

# Descriptive Statistics

Table A2: Descriptive Statistics

| Observations | Mean | Stdv | Min | Max |
| --- | --- | --- | --- | --- |
| **Suicide Rates (%)**  Entire Population 3995 | 1.45 | 0.40 | 0.14 | 3.33 |

Males 3995 2.09 0.67 0.00 5.63

Females 3995 0.85 0.36 0.00 2.58

Children 3995 0.22 0.29 0.00 2.24

Male Children 3995 0.28 0.45 0.00 3.64

Female Children 3995 0.15 0.35 0.00 3.53

Adults 3995 1.67 0.52 0.24 4.33

Male Adults 3995 2.44 0.88 0.00 7.02

Female Adults 3995 0.91 0.47 0.00 3.25

**Variables Other Than Suicide Rates**

PM_2_*_._*_5_ (AQI) 3995 50.25 12.92 17.97 99.39

Unemployment Rate (%) 3995 2.59 0.72 0.60 6.10

Table A3: Descriptive Statistics During COVID

| Observations | Mean | Stdv | Min | Max |
| --- | --- | --- | --- | --- |
| **Suicide Rates (%)**  Entire Population 164 | 1.38 | 0.37 | 0.44 | 2.42 |

Males 164 2.01 0.60 0.80 3.68

Females 164 0.79 0.37 0.00 1.75

Children 164 0.30 0.35 0.00 2.01

Male Children 164 0.34 0.55 0.00 3.64

Female Children 164 0.25 0.44 0.00 2.08

Adults 164 1.58 0.47 0.30 3.29

Male Adults 164 2.30 0.83 0.61 4.98

Female Adults 164 0.87 0.44 0.00 1.96

**Variables Other Than Suicide Rates**

PM_2_*_._*_5_ (AQI) 164 52.64 7.95 38.13 75.45

Unemployment Rate (%) 164 2.42 0.53 1.10 3.90

Table A4: Descriptive Statistics During First Wave

| Observations | Mean | Stdv | Min | Max |
| --- | --- | --- | --- | --- |
| **Suicide Rates (%)**  Entire Population 184 | 1.30 | 0.38 | 0.44 | 2.85 |

Males 184 1.96 0.66 0.64 4.05

Females 184 0.68 0.31 0.00 1.73

Children 184 0.23 0.31 0.00 1.83

Male Children 184 0.27 0.53 0.00 3.64

Female Children 184 0.18 0.37 0.00 1.87

Adults 184 1.51 0.49 0.30 3.70

Male Adults 184 2.29 0.89 0.61 5.93

Female Adults 184 0.73 0.39 0.00 1.75

**Variables Other Than Suicide Rates**

PM_2_*_._*_5_ (AQI) 184 44.13 9.71 25.55 65.58

Unemployment Rate (%) 184 2.28 0.54 0.80 3.50

Table A5: Descriptive Statistics During Second Wave

| Observations | Mean | Stdv | Min | Max |
| --- | --- | --- | --- | --- |
| **Suicide Rates (%)**  Entire Population 136 | 1.58 | 0.40 | 0.66 | 3.33 |

Males 136 2.22 0.65 0.93 4.79

Females 136 0.99 0.40 0.00 1.93

Children 136 0.39 0.45 0.00 2.24

Male Children 136 0.46 0.62 0.00 2.90

Female Children 136 0.31 0.50 0.00 2.38

Adults 136 1.79 0.54 0.51 3.90

Male Adults 136 2.47 0.86 0.52 5.65

Female Adults 136 1.11 0.51 0.00 2.30

**Variables Other Than Suicide Rates**

PM_2_*_._*_5_ (AQI) 136 42.22 13.92 17.97 75.45

Unemployment Rate (%) 136 2.45 0.58 1.10 3.90

# Air Quality Index

We use the method introduced by the U.S. Environmental Protection Agency (USEPA) to calculate the air quality index (AQI) of PM_2_*_._*_5_ (United States Protection Agency, 2018). Table [A6](#_bookmark2) illustrates the air quality index standards of PM_2_*_._*_5_ provided by the USEPA.

Table A6: Air Quality Index (AQI) scale defined by the USEPA standard

| AQI Category | Descriptor & Color | PM2*.*5  (*µg*/*m*^3^) [24-hour] |
| --- | --- | --- |
| 0 ∼50 | **Good** | 0 - 12.0 |
| 51 ∼100 | **Moderate** | 12.1 - 35.4 |
| 101 ∼150 | **Unhealth for Sensitive Groups** | 35.5 - 55.4 |
| 151 ∼200 | **Unhealthy** | 55.5 - 150.4 |
| 201 ∼300 | **Very Unhealthy** | 150.5 - 250.4 |
| 301 ∼500 | **Hazardous** | 250.5 - 500.4 |

I*_p_* = $\frac{I_{Hi}-I_{Lo}}{{BP}_{Hi}-{BP}_{Lo}}$ (*C_p_* − *BP_Lo_*) + *I_Lo._* (2)

where:

*I_p_* is the index for PM_2_*_._*_5_

*C_p_* is the truncated concentration of PM_2_*_._*_5_

*BP_HI_* is the concentration breakpoint that is greater than or equal to *C_p_ BP_Lo_* is the concentration breakpoint that is less than or equal to *C_p_*

*I_Hi_* is the AQI value corresponding to *BP_HI_ I_Lo_* is the AQI value corresponding to *BP_Lo_*

Table A7: Breakpoints for the AQI

| These Breakpoints... | . . . equal this AQI | . . . and this category |
| --- | --- | --- |
| PM2*.*5  (*µg*/*m*^3^) [24- hour] | AQI |  |
| 0 - 12.0 | 0 ∼50 | Good |
| 12.1 - 35.4 | 51 ∼100 | Moderate |
| 35.5 - 55.4 | 101 ∼150 | Unhealth for Sensitive Groups |
| 55.5 - 150.4 | 151 ∼200 | Unhealthy |
| 150.5 - 250.4 | 201 ∼300 | Very Unhealthy |
| 250.5 - 500.4 | 301 ∼500 | Hazardous |

We calculate AQI of PM_2_*_._*_5_ by using Equation [2](#_bookmark2) and Table [A7](#_bookmark3). For example, suppose you have a PM_2_*_._*_5_ value of 35.9 *µ*g/*m*^3^. First, refer to Table [A7](#_bookmark3) for the values that fall above and below your value (55.4 - 35.5). In this case, the 35.9 value falls within the index value of 101 to 150. Finally, you will get the AQI of PM_2_*_._*_5_ is obtained as follows:

$$\frac{(150-101)}{(55.4-35.5)} \left( 35.9-35.5 \right)+101=102$$

(3)

Therefore, a 24-hour PM_2_*_._*_5_ value of 35.9 *µ*g/*m*^3^ corresponds to an AQI value of 102.
